# Agromorphological Characterization and Variability Among Maize Hybrids in the Mid‐Hills of Far‐West Nepal

**DOI:** 10.1155/sci5/7227870

**Published:** 2025-12-28

**Authors:** Ram Chandra Bhatta, Akriti Risal, Asmita Shrestha, Sandesh Thapa, Mahendra Prasad Tripathi

**Affiliations:** ^1^ Department of Plant Breeding, Gokuleshwor Agriculture and Animal Science College, Tribhuvan University, Baitadi, Nepal, tribhuvan-university.edu.np; ^2^ Cooperative Extension, University of Maine, Orono, 04473, Maine, USA, maine.edu; ^3^ Department of Plant Breeding, National Maize Research Program, Chitwan, Rampur, Nepal

**Keywords:** anthesis–silking interval, cob traits, grain yield, spring maize, variability

## Abstract

The experiment was conducted using an alpha lattice design with two replications for each genotype, comprising 20 maize hybrids obtained from CIMMYT and NMRP, including two check varieties. The treatment genotypes were assessed during the spring season of 2024 at the agronomy farm of Gokuleshwor College, Baitadi. The tallest plant height of 293.298 cm was recorded in genotype RH‐12, while the shortest, 231.598 cm, was observed in RML‐95/RML‐140. The observed variation among genotypes indicated that selection can be effectively carried out based on traits such as days to 50% anthesis, days to 50% silking, anthesis–silking interval, number of rows per cob, number of grains per row, cob length, cob diameter, and grain yield. Most of the evaluated morphological traits showed a strong correlation with grain yield, depicting that indirect selection could effectively enhance yield potential. Among the hybrids, CAH 1817 stood out with the highest grain yield, followed closely by NH2226 and VH 18567. These varieties show great potential and could be strong candidates for promoting higher‐yielding maize hybrids in Nepal.

## 1. Introduction

Maize, known as the “queen of cereals” [1], is a day‐neutral C4 plant that is adaptable to diverse environments, growing successfully in hot, humid tropical and cooler temperate zones [2, 3]. It is believed that corn, or maize (*Zea mays*), originated as a wild grass in central Mexico circa 7000 years ago [4]. Native Americans improved the grain’s nutritional value [5]. A crop of global significance, maize contains around 72% carbohydrates, 10% protein, and 4% fat and has 365 kcal per 100 g [6]. Although maize is cultivated across the world, the United States, China, and Brazil are the main producers, producing about 563 million of the 717 million metric tons of maize annually [7]. Apart from being a staple grain, maize is an essential raw ingredient for many goods, including industrial alcohol, biofuels, adhesives, drinks, oils, sweeteners, and starches [8]. Millions of people in sub‐Saharan Africa depend on maize as a staple crop for their livelihoods and nutrition [9]. It is cultivated in 208 million ha in the world, producing 1241 million tons with an average productivity of 5.96 t ha^−1^, maize production increased 2.6–3.3 times faster than that of wheat or rice between 2000 and 2022, and in 2001, it surpassed rice to become the second most produced crop in the world [10].

In terms of total production and the area under cultivation, maize is the second most important crop in Nepal [11]. In the mid‐hill region of Nepal, where food insecurity remains a pressing concern, it is considered a staple that is grown from March to May, based on rainfall distribution [12]. According to [13], the area of maize cultivation in Nepal is 0.94 million ha, producing 2.97 million tons with a productivity of 3.17 t ha^−1^. Nepal’s maize production varies significantly across seven provinces. The highest production (972,073 mt) and area (284,340 ha) are found in Koshi Province, which is followed by Bagmati and Lumbini. Sudurpashchim, on the other hand, has the lowest output (101,556 mt). Madhesh has the highest productivity (3.57 mt/ha), whereas Karnali has the lowest (2.47 mt/ha), indicating regional variations in farming efficiency and agroclimatic conditions [13].

Sudurpashchim Province has the lowest maize production (101,556 mt) compared to other provinces. Baitadi, which lies in Sudurpashchim, is our study area where maize is cultivated in 0.026 million ha, producing about 0.057 million tons with an average productivity of 2.19 t ha^−1^. The demand for maize in Nepal has been steadily rising by 5% over the past 10 years, and it is predicted to increase by 4%–6% annually over the next 20 years [14].

Low maize productivity in the western hills of Nepal has traditionally been linked to the absence of high‐yielding varieties, and promoting climate‐resilient, adaptive hybrid maize genotypes along with improved agronomic practices can be a vital step toward improving the region’s food security and farmers’ incomes [15, 16]. However, productivity is further hampered by both biotic and abiotic stresses, of which insect pests impact food security and economic stability [17]. Among abiotic factors, drought stands out as one of the most devastating, limiting water availability for crops and drastically affecting their growth and yield potential [18]. Throughout the mid‐hill region of Nepal, these challenges are compounded by restricted access to improved seeds, a narrow selection of suitable hybrids, and recurring issues like gray leaf spot, northern leaf blight, temperature extremes, and waterlogging [19, 20].

Depending on the genotype and growing environment, hybrids can yield 20%–30% more than open‐pollinated varieties (OPVs) and are more responsive to fertilizer and irrigation [21]. To achieve food, feed, and hybrid seed self‐sufficiency in maize, the National Maize Research Program (NMRP) has recently sought to move the paradigm from OPVs toward hybrid maize [22]. While Rampur Hybrid‐8 and Rampur Hybrid‐10 have also been introduced to farmers in Nepal, single‐cross yellow maize hybrids like Gaurav, Rampur Hybrid‐2, Khumal Hybrid‐2, Rampur Hybrid‐4, and Rampur Hybrid‐6 are well known [23]. The purpose of this study was to describe the phenotypic characteristics of maize hybrids, find high‐yielding genotypes that are environmentally friendly, design specialized hybrids, and assist in their commercialization.

## 2. Materials and Methods

### 2.1. Study Area

**Figure 1:**
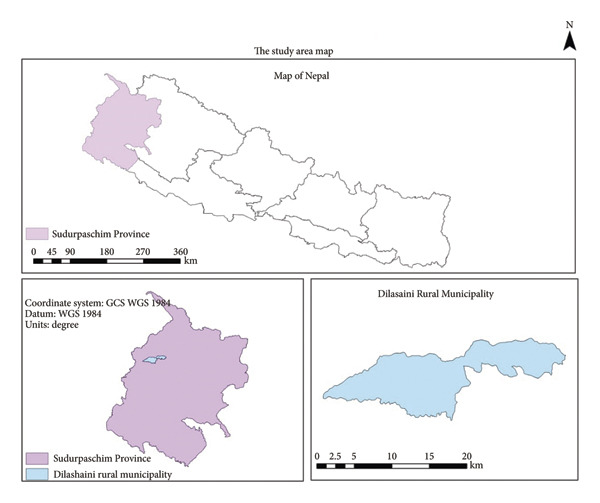
Map of Nepal showing the research area.

**Figure 2:**
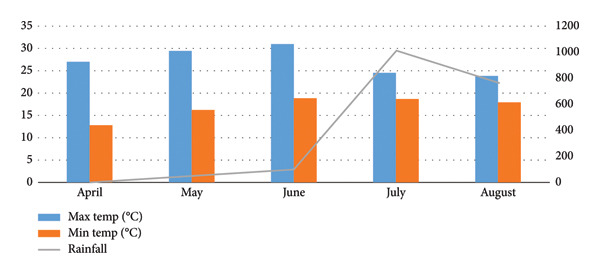
Weather data during the growing period of maize at Gokuleshwor, Baitadi (*Source:* NASA power).

The experiment was conducted at the agronomy farm of Gokuleshwor Agriculture and Animal Science College in Dilasaini, Baitadi (Figure 1), situated at an altitude of 850 m above sea level between May 1, 2024, and August 28, 2024, in the coordinates 29.6880°N latitude and 80.5494°E longitude. The average maximum and minimum temperatures during the growing period of maize were 26.8°C and 17°C, respectively, and are situated in a subtropical climate zone. The research region experienced a wide range of rainfall from 50 mm to 998 mm. The amount of rainfall was lowest in May, when planting was taking place, and highest in the pretasseling and tasseling stages. Figure 2 presents detailed agroclimatic data. Rice, wheat, and maize (standing on the field) were the previous crops grown in the experimental field.

### 2.2. Genetic Materials

Details of the genotyping sources and genotypes used in the study are shown in Table 1. The experiment consisted of 20 genotypes, seven of which were from the International Maize and Wheat Improvement Center (CIMMYT) and 13 of which were from NMRP, among which Rampur Hybrid‐12 and Rampur Hybrid‐16 were used as check varieties.

**Table 1:** Genotypes used in the experimental investigation.

SN	Name of genotypes	Source
1	RML‐150/RML‐2	NMRP
2	CML‐491/CLWH28	NMRP
3	CML‐491/CLWN 6‐377	NMRP
4	CAH 1817	CIMMYT
5	N1732‐1	CIMMYT
6	NH220	NMRP
7	CZL‐0718	CIMMYT
8	RH16	NMRP
9	N‐1731‐1	CIMMYT
10	NH2234	NMRP
11	RML‐145/Deuti	NMRP
12	RML‐150/Deuti	NMRP
13	VH 18228/V 1359‐24	CIMMYT
14	N1731‐6	CIMMYT
15	RML‐149/RL‐111	NMRP
16	RH‐12	NMRP
17	RML‐97‐2/RL‐105	NMRP
18	NH2226	NMRP
19	VH 18567/V 1777‐1	CIMMYT
20	RML‐95/RML‐140	NMRP

### 2.3. Agronomic Practices and Experimental Design

The study used an alpha‐lattice design with four blocks (10 genotypes per block) and two replications. The plot measured 4 m × 0.75 m × 2 = 6 m^2^, with a 20‐cm plant‐to‐plant and 75‐cm row‐to‐row spacing. Each genotype was sown in two consecutive rows. Deep tillage was performed to prepare the field twice, and then leveling was done. Ten tons of farmyard manure per hectare was applied. The application rates of inorganic fertilizers were 180, 60, and 40 kg·ha^−1^ of N, P2O5, and K2O, respectively. Line sowing was used to plant in two consecutive rows on May 1, 2024, and the base dose of potassium and phosphorus was applied at that time. During the initial weeding, the first nitrogen dosage was administered. Two weeding operations were conducted 25 and 40 days after sowing (DAS), respectively. At the second weeding, earthing up was completed.

### 2.4. Data Collection

For agromorphometric measures and genetic evaluations, five plants were chosen at random from each block in two replications. Except for reproductive features, all data were obtained 2 days before harvest. Agronomic traits include plant height (PHT), ear height (EHT), plant aspect (PA), husk cover (HC), and plant population per plot (PP). Cob traits include cob diameter (CD), cob length (CL), number of rows per cob (NORPC), and number of kernels per row (NOKPR). Data were collected according to the methodology explained in [24, 25]. The observed field weight (kg) and cob weight per plot were converted into grain yield (GY) (kg ha^−1^) by multiplying the conversion factor 0.8 (shelling coefficient) with 12.5% moisture adjustment (Equation (1)) [24]. The weight of 1000‐kernel samples was taken, and their moisture content was determined by using a moisture meter and converted to 12.5% moisture content (Equation (2)):

(1) GY=field weight kg×100−moisture contentplot sizem2×87.5×10,

(2) 1000−kernal weight 12.5% moisture=kernel weight×100−moisture %10012.5−.

### 2.5. Statistical Analysis

RStudio Version 4.4.1 was used for advanced statistical analysis. To determine the significance of differences between treatments, analysis of variance and Pearson correlation were computed using the package “agricolae,” followed by post hoc analysis (least significant differences) to identify mean differences, with a 5% level of significance as adopted in [26].

## 3. Results and Discussion

### 3.1. Agromorphological Characteristics of Genotypes

#### 3.1.1. Plant Architectural Traits

The analysis of variance indicated considerable differences in PHT, indicating the presence of substantial genetic variability across the genotypes examined (Table 2). There are substantial variations in PHT among hybrids/OPVs of maize [27, 28], which is in line with our current findings. According to [16], the reason for the variations in PHT among different genotypes is that environmental as well as genetic influences affect PHT. Genotype RH‐12 had a maximum PHT of 293.298 cm, while RML‐95/RML‐140 had the lowest at 231.598 cm. The study report states that the majority of genotype PHTs fell between 255 and 265 cm, with a mean PHT of 263.725 cm. Several researchers [26, 29–31] have observed PHTs within this range, which supports the current findings. The analysis of variance revealed no significant differences in EHT (*p* ≥ 0.05). The highest EHT was observed in CAH1817 (150.738 cm), while the lowest was in VH 18228/V 1359‐24 (115.866 cm), with a mean EHT of 133.34 cm. The results obtained are in line with findings by the authors in [16, 26], who discovered that maize genotypes exhibited nonsignificant EHTs. In contrast, other authors [22, 32–34] noted that EHT varied by genotype. PHT and EHT are important parameters in maize breeding because they influence selection in maize architecture. Plant density, fertilizer efficiency, water conservation, and the ability to absorb more sunlight for photosynthesis are all enhanced by optimal PHT and EHT, which raises production [35–38].

**Table 2:** Average performance of plant architectural traits.

Genotypes	PHT	EHT
RML‐150/RML‐2	259.183	138.734
CML‐491/CLWH28	270.002	149.338
CML‐491/CLWN 6‐377	259.202	136.238
CAH 1817	272.502	150.738
N1732‐1	262.183	129.534
NH220	246.183	132.034
CZL‐0718	278.802	124.438
RH16	242.683	134.734
N‐1731‐1	272.183	121.334
NH2234	267.502	141.138
RML‐95/RML 140	231.598	126.062
RML‐145/Deuti	262.317	139.766
RML‐150/Deuti	267.317	134.166
VH 18228/V 1359‐24	243.517	115.866
N1731‐6	279.298	129.962
RML‐149/RL‐111	276.998	135.962
RH‐12	293.298	137.162
RML‐97‐2/RL‐105	236.517	127.666
NH2226	289.698	137.262
VH 18567/V 1777‐1	263.517	124.666
Mean	263.725	133.34
LSD (0.05)	21.681	18.553
CV	3.897	6.595
*F*‐test	^∗∗∗^	NS
Min	211.00	106.00
Max	306.40	153.00

*Note:* The asterisk (^∗∗∗^) presented in the table indicates that the *F*‐test was highly significant at *p* ≤ 0.001, showing the presence of highly significant differences among genotypes for the respective trait (PHT). NS denotes nonsignificant differences among genotypes for the trait (EHT) at the tested probability level.

#### 3.1.2. Reproductive Traits

The analysis of variance revealed significant differences between the genotypes tested for anthesis days (AD) and silking days (SD), but no significant differences for the anthesis–silking interval (ASI). Multiple sources have reported the variability among the genotypes associated with AD and SD [28, 33, 34, 39–41], and this validates our findings. The genotype N1732‐1 displayed anthesis early, followed by RML‐149/RL‐111, NH2234, and VH 18228/V 1359‐24 (Table 3), and genotype CML‐491/CLWN revealed more days to anthesis. N1732‐1 and VH 18567/V 1777‐1 had shorter SD, while genotype CML‐491/CLWN had longer SD. VH 18228/V 1359‐24 and VH 18567/V 1777‐1 had the smallest ASI differences (0.26 days), while CML‐491/CLWN had the most (6.97 days). Pollination success is directly affected by the duration of the ASI; a shorter ASI promotes pollination, but a longer ASI might lower crop yield [42, 43]. Genotype and temperature affect reproductive traits such as AD, SD, and ASI. Higher spring temperatures shorten AD and SD because growing degree days accumulate more quickly, while lower winter temperatures lengthen these durations, causing seasonal variation [22, 32, 40] and further influencing genotype [44]. The authors in [45] indicated that the days to flowering differed by 2 weeks between the early and late genotypes, resulting in a 1‐month difference in maturity between the early and late maturing genotypes. Spring maize experienced slower early development due to the low starting temperature until mid‐April; therefore, it took longer to attain tasseling and silking than summer maize [46].

**Table 3:** Performance of reproductive traits.

Genotypes	AD	SD	ASI
RML‐150/RML‐2	65.146	68.015	3.24
CML‐491/CLWH28	68.231	69.472	1.97
CML‐491/CLWN	68.231	74.472	6.97
CAH 1817	68.731	73.972	5.97
N1732‐1	62.646	64.015	1.74
NH220	64.146	69.015	5.24
CZL‐0718	64.231	67.972	4.47
RH16	65.646	70.015	4.74
N‐1731‐1	66.146	67.515	1.74
NH2234	63.231	67.472	4.97
RML‐95/RML 140	67.769	71.528	3.03
RML‐145/Deuti	67.354	68.985	1.26
RML‐150/Deuti	63.854	68.485	4.26
VH 18228/V 1359‐24	63.354	67.985	0.26
N1731‐6	64.769	65.028	0.47
RML‐149/RL‐111	62.769	65.528	2.03
RH‐12	65.269	68.028	2.03
RML‐97‐2/RL‐105	65.854	69.485	3.26
NH2226	65.769	69.028	2.53
VH 18567/V 1777‐1	63.854	64.485	0.26
Mean	65.350	68.525	3.175
LSD (0.05)	3.425	4.195	3.590
CV	2.484	2.902	53.595
*F*‐test	^∗∗^	^∗∗∗^	NS
Min	60.000	62.000	0.000
Max	72.000	76.000	9.000

*Note:* The asterisk symbols associated with the *F*‐test indicate the level of statistical significance among genotypes for the reproductive traits. ^∗∗^Denotes moderately significant differences at *p* ≤ 0.01, while ^∗∗∗^ denotes highly significant differences at *p* ≤ 0.001. NS indicates nonsignificant differences among genotypes at the tested probability level. Accordingly, genotypes differed significantly for anthesis date (AD) and highly significantly for silking date (SD), whereas no significant variation was observed for anthesis–silking interval (ASI).

#### 3.1.3. Cob Characteristics

Analysis of variance revealed substantial differences across genotypes for the number of grains per row (NOGPR), NORPC, and CD, all of which positively influenced GY, as supported by the findings in [28, 32, 33, 47]. The CD varied from 3.618 (RH16) to 4.763 (CAH 1817), with 4.202 cm on average. CL ranged from 16.017 (CML‐491/CLWN) to 20.843 (N‐1731‐1), with an average of 18.783 cm. We analyzed the NOGPR and the NORPC in the genotypes. The maximum number of NORPC was reported in CAH 1817 (16.702) and the lowest in NH2234 (12.702). Similarly, the NOGPR was the highest in VH 18567/V 1777‐1(39.279) and the lowest was in RH16 (25.571). NORPC and NOGPR both increase GY; the more rows per cob and grains per row, the greater the GY, and vice versa [30]. Report from hybrid maize evaluation in Nepal showed that the mean number of kernel rows per cob and grains per row ranged from 14 to 16 and 28 to 35, respectively [29, 30, 33, 48]. Compared to published data [32, 33, 48], we discovered that the mean performance of genotypes fell within a similar range. However, the individual performance of some hybrid genotypes such as CAH 1817, NH2226, CML‐491/CLWH 28, and RML‐150/RML‐2 was superior and recorded comparatively with higher GYs along with cob traits such as greater CL, CD, and kernel number per row.

#### 3.1.4. Thousand‐Grain Weight and GY

The study showed that the genotypes differed in grain production and thousand‐grain weights, and the same outcome was also attained in [27, 28, 39, 41]. The highest thousand‐grain weight was found in N1731‐1 (0.407 kg), N1731‐6 (0.395 kg), and NH2226 (0.384 kg), while the lowest thousand‐grain weight was achieved in RML‐95/RML 140 (0.271 kg), with a mean thousand‐grain weight of 0.332 kg. The mean GY per hectare was 6.794 tons/ha. CAH 1817 had the highest GY at 7.659 ton/ha, followed by NH2226 (7.556 ton/ha), VH 18567/V 1777‐1 (7.548 ton/ha), and CML‐491/CLWH28 (7.540 ton/ha) (Table 4). Since GY depends on yield‐controlling features and directly affects farmers’ productivity and profitability, it is the primary economic attribute for improvement in breeding projects [26]. Several authors [22, 31, 41, 45, 49] demonstrated that hybrid maize’s grain output varies significantly, allowing genotypes to be chosen based on how well they perform. According to [22], the GY of heat‐resilient maize varied between 3.5 and 10.35 tons per hectare. Similarly, the authors in [33] discovered that the range of GY was 8.98–10.3 t/ha, while the authors in [22] observed that GY ranged from 3.88 to 10.11 ton/ha and had statistical significance.

**Table 4:** Mean performance and analysis of variance of yield and cob characteristics.

Genotypes	Number of cobs	Number of kernels per row	Number of rows per cob	Cob length	Cob diameter	Test weight	Grain yield
RML‐150/RML‐2	32.486	33.871	15.549	19.243	4.629	0.358	7.504
CML‐491/CLWH 28	40.567	37.794	14.502	17.967	4.138	0.298	7.541
CML‐491/CLWN	35.567	28.344	15.302	16.017	4.363	0.312	7.122
CAH 1817	34.067	26.344	16.702	17.767	4.763	0.377	7.659
N1732‐1	33.486	35.671	14.949	20.193	4.078	0.346	7.492
NH220	35.986	29.371	14.149	18.593	3.962	0.303	6.363
CZL‐0718	25.067	33.944	14.202	19.417	4.127	0.368	6.448
RH16	26.986	25.571	15.149	16.643	3.618	0.292	4.030
N‐1731‐1	31.486	27.521	13.949	20.843	4.123	0.407	6.809
NH2234	34.567	30.694	12.702	18.417	3.729	0.366	6.975
RML‐95/RML 140	36.933	31.356	14.498	18.983	4.129	0.271	6.548
RML‐145/Deuti	32.014	36.129	14.051	19.907	4.207	0.340	7.123
RML‐150/Deuti	36.014	33.029	13.451	17.457	4.333	0.329	7.266
VH 18228/V 1359‐24	30.014	32.329	14.851	19.257	4.454	0.335	7.102
N1731‐6	34.933	28.156	14.098	18.693	4.038	0.395	6.830
RML‐149/RL‐111	29.933	35.856	15.098	16.183	4.552	0.273	6.776
RH‐12	30.433	37.456	14.898	19.983	4.288	0.316	6.656
RML‐97‐2/RL‐105	27.514	36.854	12.801	19.057	3.991	0.287	4.533
NH2226	30.933	34.856	13.898	20.483	4.253	0.384	7.557
VH 18567/V 1777‐1	31.514	39.279	14.751	20.557	4.259	0.278	7.548
Mean	32.525	32.721	14.478	18.783	4.202	0.332	6.794
LSD (0.05)	9.647	5.582	0.907	2.999	0.367	0.052	1.528
CV	14.058	8.085	2.971	7.567	4.142	7.361	10.658
*F*‐test	NS	^∗∗∗^	^∗∗∗^	NS	^∗∗∗^	^∗∗∗^	^∗∗^
Min	23.000	22.800	12.000	15.200	3.482	0.242	3.254
Max	44.000	39.800	17.200	22.000	4.830	0.452	8.812

*Note:* The asterisk symbols associated with the *F*‐test indicate the level of statistical significance among genotypes for the reproductive traits.

Abbreviation: NS = nonsignificant.

∗Significant at *p* ≤ 0.05.

∗∗Moderately significant at *p* ≤ 0.01.

∗∗∗Highly significant at *p* ≤ 0.001.

### 3.2. Correlation Analysis

The correlation study shows several noteworthy correlations between GY and various yield‐associated characteristics (Table 5). GY was positively and significantly correlated with PHT and CD, suggesting that taller plants are likely to yield more [50]. Test weight (TW) also exhibited a moderate positive correlation with GY (0.42^∗∗^), suggesting that heavier grains contribute to better yield outcomes, which was also reported in [44]. Traits like EHT, CL, NOGPR, and NORPC showed weak positive correlations (0.30, 0.28, 0.24, and 0.23, respectively), indicating only a modest impact on GY. In contrast, there were adverse correlations between yield and reproductive parameters such as the ASI, AD, and SD, indicating that genotypes with shorter intervals between anthesis and silking and early flowering are likely to yield more. Similar result was observed in [26], which supports our finding. Furthermore, the highly substantial positive correlation (0.55^∗∗∗^) between PHT and EHT highlights the merit of plant architectural traits. The CL and NOGPR have a strong positive correlation (0.52, ^∗∗∗^), suggesting that longer cobs can sustain more grain per row. As a result, CL is a crucial characteristic for increasing yield potential. Overall, characteristics including PHT, grain weight, CD, and minimum ASI were shown to be important markers for choosing high‐yielding genotypes.

**Table 5:** Correlation analysis between yield and yield‐governing traits.

	PHT	EHT	NORPC	NOGPR	CL	CD	AD	SD	ASI	TW	GY
Plant height	1	0.55^∗∗∗^	0.22ns	0.29ns	0.22ns	0.35^∗^	−0.32^∗^	−0.43^∗^	−0.35^∗^	0.43^∗∗^	0.54^∗∗∗^
Ear height		1	0.34^∗^	0.23ns	−0.06ns	0.32^∗^	−0.11ns	−0.16ns	−0.15ns	0.00ns	0.30ns
NORPC			1	0.04ns	−0.11ns	0.64^∗∗∗^	−0.05ns	−0.05ns	−0.02ns	−0.10ns	0.28ns
NOGPR				1	0.52^∗∗∗^	0.27ns	−0.37^∗^	−0.49^∗∗^	−0.38^∗^	−0.15^∗^	0.24ns
CL					1	0.02ns	−0.14ns	−0.34^∗^	−0.43^∗∗^	0.39^∗^	0.23ns
CD						1	−0.15ns	−0.13ns	−0.04ns	0.18ns	0.56^∗∗∗^
AD							1	0.84^∗∗∗^	0.16ns	−0.07ns	−0.15ns
SD								1	0.67^∗∗∗^	−0.19ns	−0.34^∗^
ASI									1	−0.25ns	−0.40^∗∗^
TW										1	0.42^∗∗^
GY											1

*Note:* The symbols are defined as follows: ^∗^indicates significance at *p* ≤ 0.05, ^∗∗^indicates moderate significance at *p* ≤ 0.01, ^∗∗∗^indicates high significance at *p* ≤ 0.001. The “+” and “−” symbols represent positive and negative correlations, respectively, and the value “1” indicates perfect correlation.

Abbreviation: NS, nonsignificant correlations.

## 4. Conclusion

There was a significant variation observed among the maize hybrid genotypes for agromorphological traits, indicating considerable potential for selection‐based genetic improvement. One of the main criteria used to select superior hybrids was high GY. The results of this study showed that maize hybrids, including CAH 1817, NH2226, and VH 18567/V 1777‐1, performed better or on par with conventional checks, indicating their potential for increased productivity in far‐west Nepal’s mid‐hill settings. Therefore, these hybrids are recommended for further evaluation through multilocation, multiyear, and farmer‐managed field trials to validate their performance and consider them for future large‐scale recommendation, release, or commercialization.

## Conflicts of Interest

The authors declare no conflicts of interest.

## Author Contributions

Ram Chandra Bhatta, Akriti Risal, and Asmita Shrestha contributed equally to this work.

## Funding

The project was partially funded by Gokuleshwor Agriculture and Animal Science College, Baitadi, Nepal, under partial fulfillment of the RPS which includes the availability of field site and assistance is field operations.

## Data Availability

The data used to support the findings of the study are included within the article.

## Appendix A Limitations of This Study

Due to funding constraints, this study was only conducted for 1 year and one season.
